# Multispecies probiotic administration reduces emotional salience and improves mood in subjects with moderate depression: a randomised, double-blind, placebo-controlled study

**DOI:** 10.1017/S003329172100550X

**Published:** 2023-06

**Authors:** Rita Baião, Liliana P. Capitão, Cameron Higgins, Michael Browning, Catherine J. Harmer, Philip W. J. Burnet

**Affiliations:** 1 Department of Psychiatry, University of Oxford, Warneford Hospital, Oxford, OX3 7JX, UK; 2 Oxford Health NHS Foundation Trust, Oxford, UK

**Keywords:** Bifidobacteria, emotional memory, Lactobacilli, mood disorder

## Abstract

**Background:**

The potential antidepressant properties of probiotics have been suggested, but their influence on the emotional processes that may underlie this effect is unclear.

**Methods:**

Depressed volunteers (*n* = 71) were recruited into a randomised double-blind, placebo-controlled study to explore the effects of a daily, 4-week intake of a multispecies probiotic or placebo on emotional processing and cognition. Mood, anxiety, positive and negative affect, sleep, salivary cortisol and serum C-reactive peptide (CRP) were assessed before and after supplementation.

**Results:**

Compared with placebo, probiotic intake increased accuracy at identifying faces expressing all emotions (+12%, *p* < 0.05, total *n* = 51) and vigilance to neutral faces (mean difference between groups = 12.28 ms ± 6.1, *p* < 0.05, total *n* = 51). Probiotic supplementation also reduced reward learning (−9%, *p* < 0.05, total *n* = 51), and interference word recall on the auditory verbal learning task (−18%, *p* < 0.05, total *n* = 50), but did not affect other aspects of cognitive performance. Although actigraphy revealed a significant group × night-time activity interaction, follow up analysis was not significant (*p* = 0.094). Supplementation did not alter salivary cortisol or circulating CRP concentrations. Probiotic intake significantly reduced (−50% from baseline, *p* < 0.05, *n* = 35) depression scores on the Patient Health Questionnaire-9, but these did not correlate with the changes in emotional processing.

**Conclusions:**

The impartiality to positive and negative emotional stimuli or reward after probiotic supplementation have not been observed with conventional antidepressant therapies. Further studies are required to elucidate the significance of these changes with regard to the mood-improving action of the current probiotic.

## Introduction

There is now compelling evidence for a link between the enteric microbiota and brain function and supplements containing live bacteria (probiotics) or dietary fibres that grow intrinsic beneficial gut bacteria (prebiotics) are considered as ‘psychobiotics’ if they confer a mental health benefit to the host when ingested in appropriate amounts (Dinan, Stanton, & Cryan, 2013; Sarkar et al., 2016). Administration of the probiotic *Bifidobacterium longum* 1714 is reported to have a pro-cognitive effect in healthy volunteers (Allen et al., 2016), alleviate stress and anxiety in individuals exposed to a controlled stressor (Wang, Braun, Murphy, & Enck, 2019) and even modulate sleep parameters in students exposed to examination stress (Moloney et al., 2020). Specific *Lactobacilli* strains also demonstrate anxiolytic and antidepressant properties (Chong et al., 2019), and improve sleep (Nishida, Sawada, Kuwano, Tanaka, & Rokutan, 2019). However, a recent systematic review and meta-analysis of probiotics in clinical populations of major and co-morbid depression revealed that multispecies probiotics (consisting of both *Bifidobacteria* and *Lactobacilli* strains) are better than single strains for improving mood (Liu, Walsh, & Sheehan, 2019). In spite of these encouraging findings however, the psychological mechanisms underlying the psychotropic effects of probiotics remain elusive, and may be different to those influenced by contemporary pharmacotherapies. In this instance, understanding the influence of probiotics on emotions, may suggest how they should be most effectively combined with other interventions.

Negative affective biases in emotional processing are believed to have a key role in the aetiology and pathophysiology of depression. Depressed individuals are more likely to interpret, focus on and remember negative compared to positive emotional cues in self-relevant neuropsychological tasks (Harmer, Goodwin, & Cowen, 2009a; Harmer et al., 2009b). Depression has also been associated with anhedonia, a loss of pleasure in response to receipt of reward. People with depression fail to develop a response bias towards favourable results in reward learning tasks (Pizzagalli, Iosifescu, Hallett, Ratner, & Fava, 2008; Walsh, Browning, Drevets, Furey, & Harmer, 2018), and there is evidence that conventional antidepressants may increase subjects' response to choices associated with positive outcomes (Scholl et al., 2017). Therefore, recent theories suggest that over time, positive changes in emotional bias and cognitive/reward processing contributes to improved mood.

We have reported that the intake of a prebiotic (a dietary fibre that grows indigenous beneficial gut bacteria) improved attention to positive information and reduced awakening salivary cortisol in healthy volunteers (Schmidt et al., 2015). However, subjective mood ratings were not affected, probably because volunteer assessments were relatively insensitive given that depression scores were low on average and participants were excluded if they met the criteria for depression or anxiety. Therefore, the true psychotropic actions of nutritional supplements are more likely to be observed in the presence of a pre-existing deficit, and one study concluded that more convincing evidence to support the use of gut bacteria-based therapies for mood dysfunction will be provided from investigations of probiotics in depression (Liu et al., 2019).

The aim of the current study was to test if a daily, 4-week intake of a multispecies probiotic improved emotional processing and reward learning (primary outcomes) in subjects with untreated moderate depression. This population was chosen because first, people with depression are more likely to have aberrant emotional processing (Godlewska & Harmer, 2021) and so changes after probiotic intake could be more readily detected; and second, moderately depressed subjects were less likely to be undergoing treatment, and so the confounding effects of medication or psychotherapy would be avoided. Our hypothesis was that probiotic intake would reduce bias to negative emotional cues and/or increase bias to positive cues compared to placebo.

Since this investigation was exploratory, we also examined the effect of the supplement on implicit memory and auditory verbal learning to test the pro-cognitive potential of the probiotic. In view of the aforementioned evidence for probiotics improving sleep in stressed individuals, we also measured sleep parameters with actigraphy, particularly given sleep disturbance is common in depression (Murphy & Peterson, 2015). Finally, the concentrations of salivary cortisol and blood C-reactive peptide (CRP) were measured as indices of the neuroendocrine stress response and immune reactivity, respectively, to provide further mechanistic information. The cortisol awakening response has been shown to reduce after the intake of prebiotics as mentioned above, while circulating CRP levels in subjects with major depression, decrease following probiotic supplementation (Akkasheh et al., 2016).

## Methods

### Participants

A power calculation to determine the sample size was based on data showing that acute antidepressant administration reduced accuracy to detect fearful faces- or poorer performance in detecting negative emotions- in healthy volunteers, with an effect size of 1.09 (Harmer, Shelley, Cowen, & Goodwin, 2004). For the current study, the minimum sample size required to detect changes in accuracy (difference between two independent means: two-tailed, *α* = 0.05, effect size = 1.09, power = 0.95) was calculated to be *n* = 23 per group. Given the possibility of drop-out (conservatively estimated as 20%), the recruitment target was inflated and rounded off to a total of 60 participants.

A summary of recruitment is summarised in a flow diagram based on CONSORT (online Supplementary Fig. S1). The study drop out was approximately 8%, which allowed the target sample size of 46 to be met before 60 participants were recruited, and resulted in a total sample size of 51. Collection of data from the last nine participants was prevented because of ineligibility (*n* = 1), dropout owing to personal or undisclosed reasons (*n* = 6), and side effects (*n* = 2). Although an adequate number of participants had completed the primary emotional processing/cognitive outcomes at this point, ethical permission was granted to recruit an additional 20 participants to further increase the power of biological measures. However, owing to the COVID-19 pandemic, these subjects completed only those outcomes that could be easily performed remotely (PHQ-9 and provision of saliva samples via post for cortisol awakening response data).

All participants were recruited from the local area and neighbouring cities, using posters, web advertisements and social media, and were pre-screened to ensure they scored within the range of 5 and 19 on the Patient Health Questionnaire-9 [PHQ-9; (Kroenke, Spitzer, & Williams, 2001)] for mild to moderate depression. All interviews were performed by Dr Rita Baiao, a trained clinical psychologist, who was supported when necessary by a psychiatrist. Exclusion criteria included: current psychiatric disorder (except for depression and anxiety), substance misuse, current intake (or intention to take) any medication that may affect the outcomes, intake of probiotics or prebiotics, major medical disorders (including diseases affecting the human gastrointestinal tract), a body mass index (BMI) outside the range of 18–30 kg/m^2^, current psychological therapy, recent significant changes in diet, dyslexia, and prior exposure to task battery. The Structural Clinical Interview for the DSM-IV Axis I Disorders [SCID-I, (First, Spitzer, & Williams, 2002)] was used to exclude for current/past comorbid psychiatric disorders, and to exclude cases with a need for immediate treatment or suicide risk.

### Experimental design

**Fig. 1.**
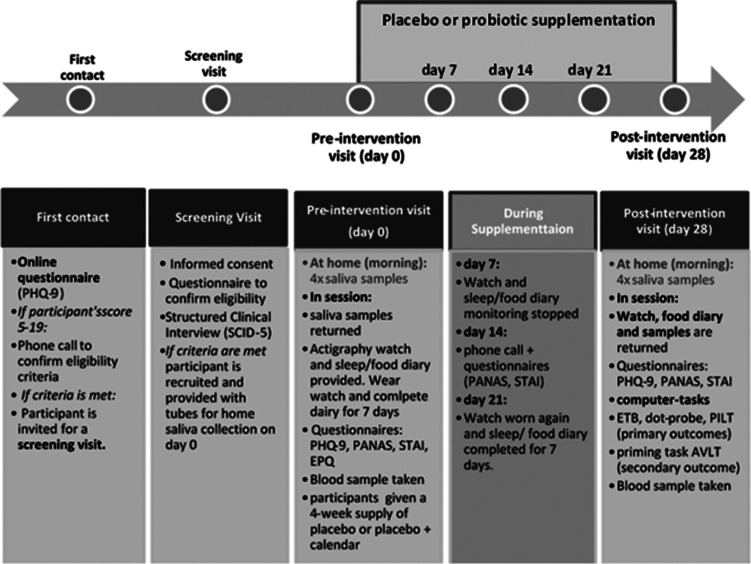
Schematic of the experimental procedure to test the effects of a 28-day intake of placebo or a multispecies probiotic on emotional processing in people with moderate depression. *PHQ-9*, Patient Health Questionnaire-9; *SCID-5*, Structural Clinical Interview for the DSM-V Axis I Disorders; *PANAS*, Positive And Negative Affective Scale; *STAI*, State/Trait Anxiety Inventory; *EPQ*, Eysenck personality Questionnaire; *ETB*, Emotional Processing Test Battery; *PILT*, Probabilistic instrumental learning task; *AVLT*, Auditory verbal learning task.

A schematic of the experimental design is shown in Fig. 1. This study was designed as a randomised, parallel, double-blind, placebo-controlled intervention (1:1 allocation), which was approved by the University of Oxford Central University Research Ethics Committee (ref: R58085/RE001), and registered with ClinicalTrials.gov (NCT03801655). Participants were stratified for gender and randomly allocated to either placebo or probiotic (see online Supplementary information). The probiotic (Bio-Kult^®^ Advanced, ADM Protexin Ltd), consisted of 14 species of bacteria, (*Bacillus subtilis PXN^®^ 21, Bifidobacterium bifidum PXN^®^ 23, Bifidobacterium breve PXN^®^ 25, Bifidobacterium infantis PXN^®^ 27, B. longum PXN^®^ 30, Lactobacillus acidophilus PXN^®^ 35, Lactobacillus delbrueckii ssp. bulgaricus PXN^®^ 39, Lactobacillus casei PXN^®^ 37, Lactobacillus plantarum PXN^®^ 47, Lactobacillus rhamnosus PXN^®^ 54, Lactobacillus helveticus PXN^®^ 45, Lactobacillus salivarius PXN^®^ 57, Lactococcus lactis ssp. lactis PXN^®^ 63, Streptococcus thermophilus PXN^®^ 66*), encapsulated at 2 × 10^9^ CFU/capsule with a cellulose bulking agent in a vegetable capsule (hydroxypropyl methylcellulose). Placebo capsules were matched in size, shape, colour and composition, except for the live bacteria. A 4 week daily supply of capsules was provided to volunteers in blister packs at the beginning of the study. Participants were asked to take four capsules in the morning each day with food.

Side effects were measured at the post-intervention session according to participant's report, and participants were asked to guess which group they were in. Compliance was assessed by counting the number of blisters returned by participants at the end of the study, and by checking the calendar where participants registered their capsule intake.

#### Subjective questionnaire measures

At the beginning of the study (day 0), participants completed the: PHQ-9, to assess mood; Positive and Negative Affect Scale [PANAS, (Watson, Clark, & Tellegen, 1988)] to assess affect; Spielberger State-Trait Anxiety Inventory [STAI, (Spielberger & Gorsuch, 1983)] to assess anxiety; and Eysenck Personality Questionnaire (Eysenck & Eysenck, 1975) to assess personality traits. PHQ-9 and STAI were repeated at the end of supplementation (day 28), and the PANAS was repeated on day 14 of supplementation, and again on day 28. On day 0 and day 28, participants completed a 7 day food diary with information on food groups, alcohol, water and caffeine ingestion (online Supplementary Fig. S2).

#### Emotional processing

The primary outcomes [emotional test battery (ETB)], dot-probe task and tests of reward learning) and secondary outcomes (implicit memory and auditory verbal memory) were performed by participants at the end of the supplementation (day 28) in the Department of Psychiatry, University of Oxford. All computer-based tasks were only performed on the last day of supplementation owing to concerns of potential learning effects when tasks are repeated (Harmer, Cowen, & Goodwin, 2011). The ETB comprises five tasks measuring a participant's bias towards positive or negative valence facial expressions and words, and consisted of the facial expression recognition task (FERT), emotional categorisation (ECAT), attentional dot-probe, emotional recall (EREC), and emotional recognition memory (EMEM) task. Details of all tasks are presented in online Supplementary information.

#### Probabilistic instrumental learning task (PILT)

Details of this reward task are presented in online Supplementary information. The task measures the degree to which participants learn to select the most advantageous outcome from the win and loss trials, which is parameterised as the proportion of trials in the win/loss conditions in which the advantageous option (i.e. associated with 0.7 win outcomes or 0.7 no loss outcomes) was chosen.

#### Cognitive tasks

Participants performed tests of implicit learning (Priming Task) and verbal memory (auditory verbal learning task, AVLT). Detailed methods are provided in online Supplementary information. The *priming task* measured implicit memory, where prior exposure to a stimulus influences responses to the next stimulus (Klinge et al., 2018). Differences in the reaction times from the experimental and control conditions were calculated. The *AVLT* measured verbal memory and was run as previously described (Murphy, Wright, Browning, Cowen, & Harmer, 2020). The number of correct words in each trial were analysed.

#### Sleep and activity

Sleep and activity were measured objectively using actigraphy (MotionWatch8, CamNTech, Ltd, Cambridgeshire, UK) during week 0 and week 4 of intervention. Actual sleep time, sleep efficiency (%), sleep latency, wake bouts, mean wake bout, immobile minutes, total activity (during the assumed sleep period), and mean activity/30 s epoch were analysed as previously described (Maurer et al., 2020) and detailed in online Supplementary information. Participants also recorded the times they went to bed and woke up (see online Supplementary Fig. S2).

#### Biological measures

To analyse salivary cortisol, participants provided four saliva samples collected at home (every 15 min from waking up) before pre- and post-intervention sessions. Saliva was centrifuged (2000 ***g*** for 10 min) and stored in a −80 °C freezer. Cortisol concentrations were determined using a commercial cortisol enzyme-linked immunosorbent assay (ELISA) kit and following manufacturer's instructions (Salimetrics, UK). Blood was obtained from participants by venepuncture, at pre- and post-intervention sessions keeping the time of day similar for each session. Samples for plasma (10 ml) were collected in appropriate vacutainer tubes and centrifuged to render samples acellular, and stored at −80 °C. The concentration of plasma CRP was determined using a commercial ELISA kit (Abcam, UK).

### Statistical analysis

All analyses were performed using IBM SPSS Statistics for Windows (version 24.0; IBM Corp., Armonk, NY). Data are presented as estimated marginal means ± s.e.m. and were analysed with repeated measures, where group (probiotic and placebo) was the between-subject factor. Within-subject factors were ‘time’ (pre- *v.* post-intervention) for questionnaires and sleep measures, and ‘emotion/valence’ for the ETB, PILT and dot-probe task. For the AVLT, the within-subject factor in the analysis of List A was the five trials, and for the interference trial, ListA/trial 1 and List B were within-subject factors. Regarding cortisol analysis, the time-point of saliva collection (0, 15, 30, 45 after waking up) and day of sampling (pre- *v*. post-treatment) were the within-subject factors. Significant interactions were explored using pair-wise comparisons. Statistical significance (*p* < 0.05) and marginal differences (*p* < 0.10) were reported. The effect size was estimated using *ηp*^2^. Corrections for the use of multiple tasks were not made as they were considered too stringent for the between-subject design of the study. This is an approach that has been accepted for our other studies with the same design where a change in emotional processing was the primary outcome (e.g. Capitão et al., 2020).

Correlations between subjective ratings, results from the ETB, cognitive tasks and reward learning were explored with bivariate Pearson's *r* analysis. Participants' treatment guess and their assigned group were analysed by the χ^2^ test. The number of capsules taken in the placebo and the probiotic group was compared with a *t* test.

## Results

### Baseline characteristics and compliance

Seventy-one participants (26 males, 45 females) aged 18–55 years with moderate depression were randomly allocated to placebo (*n* = 36, 13 male), or probiotic groups (*n* = 35, 13 male). Subjects' demographic details are presented in Table 1. Empty blister packets were returned by the participants at the end of the treatment indicated that compliance levels did not differ between groups (number of tablets returned: *t* test, *p* = 0.40), and there was no association between group and treatment guess (χ^2^ = 0.089, *p* = 0.765).

**Table 1.** Demographic details and baseline characteristics of all participants and group assignment after randomisation

	Min-Max	Whole sample (*N* = 71)		Probiotic (*N* = 35)		Placebo (*N* = 36)	
		Mean	s.d.	Mean	s.d.	Mean	s.d.
Age	18–55	28.80	8.93	27.94	6.99	29.64	10.52
BMI	18.5–30	23.00	2.90	23.03	3.00	22.97	2.84
EPQ-N	–	15.97	4.25	15.60	4.94	16.34	3.45
EPQ-P	–	3.32	2.37	3.17	2.48	3.48	2.29
EPQ-E	–	10.15	5.30	9.40	5.07	10.93	5.50
		*N*	%	*N*	%	*N*	%
Gender	Male	26	36.6	13	37.1	13	36.1
	Female	45	63.4	22	62.9	23	63.9
Ethnicity	Caucasian	58	81.7	29	82.9	29	80.6
	Black	1	1.4	0	0	1	2.8
	Hispanic	1	1.4	0	0	1	2.8
	Asian	10	14.1	5	14.3	5	13.9
	Mixed	1	1.4	1	2.9	0	0
Education	Secondary	22	31	13	37.1	9	25
	Undergraduate	16	22.5	7	20.0	9	25
	Postgraduate	33	46.5	15	42.9	18	50

BMI, body mass index; EPQ, Eysenck Personality Questionnaire (–N, neuroticism; –P, psychoticism; –E, extrovert).

### Effects of intervention (probiotic *v*. placebo)

- Emotional processing

All ETB and dot-probe data are summarised in online Supplementary Table S1.

#### FERT

**Fig. 2.**
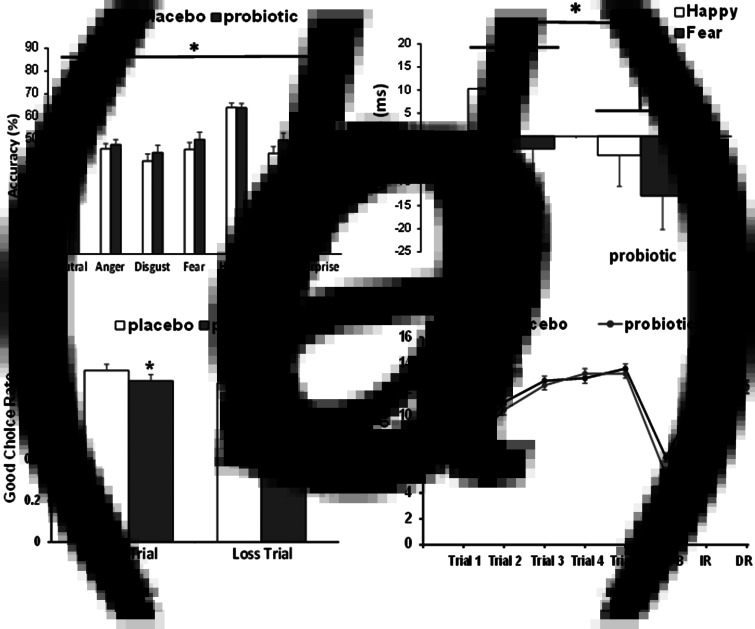
The effect of a multispecies probiotic on the performance of emotion and cognitive measures after 28 days supplementation. (*a*) Accuracy at identifying facial expression of all emotions was significantly greater after probiotic intake (*n* = 26) than placebo (*n* = 25). Bar indicates the effect of the group (*p < 0.05). (*b*) Probiotic intake reduces attentional vigilance to positive and negative emotions in the dot-probe task. In the unmasked condition, there was a main effect of group (*p < 0.05) where probiotic supplemented subjects (*n* = 26) displayed less attention to happy and fearful emotions compared to placebo (*n* = 25). (*c*) The influence of the placebo or probiotic on the performance of the PILT after supplementation. There was an overall effect of group (*p < 0.05), where the good choice rate for both the win and loss trials were reduced by the probiotic (*n* = 26) compared to placebo (*n* = 25). (*d*) AVLT learning curves after placebo or probiotic supplementation. There was a significant group × trial interaction when comparing the recollection of List B words with List A/Trial 1 words. The recall of fewer List B words by the probiotic group was indicative of proactive interference. Probiotic intake did not affect List A recall (Trials 1–5), immediate recall (IR) or delayed recall (DR). *p < 0.05, group × List B/List A -Trial 1 interaction.

*Accuracy*: In the absence of a significant emotion × group interaction (*F*_6,294_ < 2.0, *p* > 0.05), a main effect of group was observed (*F*_1,49_ = 4.747, *p* = 0.034, *ηp*^2^ = 0.088), where the probiotic group was more accurate than placebo at classifying faces across emotions (number of overall correct classifications: placebo = 51.8 ± 1.41, probiotic = 56.2 ± 1.43; Fig. 2*a*).

*Reaction time* (*RT*): Analysis did not reveal a significant effect of group (placebo = 1776.3 ± 73.6 ms, probiotic = 1795.1 ± 75.1 ms, *p* > 0.05) nor a group × emotion interaction (*F*_1,49_ < 2.0, *p* > 0.050).

*Misclassifications:* There were no significant effects of group (number of incorrect classifications: placebo = 18.6 ± 0.95, probiotic = 16.5 ± 0.95), or interaction between treatment and group (*F*_1,49_ < 2.0, *p* > 0.050).

#### ECAT, EREC, EMEM

There were no significant effects of group or group × valence interaction for either accuracy or RT in these tasks (*F*_1,49_ < 2.0, *p* > 0.050).

#### DOT-PROBE

There were no significant interaction effects with valence or masking (all *p* > 0.2). A main effect of group in the unmask condition (*F*_1,49_ = 4.307, *p* = 0.043, *ηp*^2^ = 0.081) was found, with the probiotic group being less vigilant to positive and negative emotional cues relative to neutral stimuli (placebo = +4.12 ± 2.67 ms, probiotic = −3.81 ± 2.72 ms; Fig. 2*b*). The groups did not differ in their overall accuracy. There were no significant effects in the marked condition (online Supplementary Table S1).

- PILT

There was a main effect of group (*F*_1,49_ = 4.14, *p* = 0.047, *ηp*^2^ = 0.078) with participants taking the probiotic showing a reduced tendency to select the advantageous option from both win and loss trials (Fig. 2*c*). All subjects' win correct choices were significantly greater than loss choices (win = 0.80 *v.* loss = 0.72, *p* = 0.001). No interactions were observed.

- Cognition

AVLT – data are presented in Fig. 2*d*. All participants' recall of List A words improved across the five acquisition trials (*F*_4,196_ = 173.26, *p* < 0.001), in the absence of a group × trial interaction. However, in the interference trial, there was a significant group × trial interaction (*F*_1,48_ = 4.29, *p* = 0.044, *ηp*^2^ = 0.082), where the probiotic group recalled fewer words in List B compared to List A/Trial 1 (*F*_1,48_ = 6.37, *p* = 0.015, *ηp*^2^ = 0.117), and therefore exhibited increased susceptibility to proactive interference. There was no difference between groups in the number of words accurately recalled after a short and long delay, nor in the recognition task.

Priming task – No significant differences between treatment groups were found (online Supplementary Table S1, *F*_1,49_ < 2.0, *p* > 0.050).

- Sleep and activity

Sleep data from subjects supplemented with either placebo or probiotic are summarised in online Supplementary Table S2. A significant total activity × group interaction for total activity was also found (*F*_1,47_ = 5.454, *p* = 0.024). The probiotic group had greater total activity after 4 weeks compared to the start of the study, though follow up analysis did not reveal significant differences (*F*_1,47_ = 2.930, *p* = 0.094). A trend interaction effect was also found for mean activity/epoch (*F*_1,47_ = 3.604, *p* = 0.064). There were no effects of treatment on the other objective sleep variables, including sleep latency. Data on food intake, which were logged in the same diary, are summarised in online Supplementary Table S3. No significant interaction between food item, time or group were observed.

1. Biological measures

There was no significant time point × group interaction (*F*_7,462_ < 2.0, *p* > 0.05) or main effect of group for awakening salivary cortisol concentrations, in spite of an apparent lower response in the probiotic group after 4 weeks (Fig. 2*a*, *b*, online Supplementary Table S4). Analysis of serum CRP did not show a group × time point interaction or main effects of time or group (Fig. 2*c*, online Supplementary Table S4).

- Subjective ratings

Participants' subjective mood, anxiety and affect ratings are presented in Table 2. There was a significant time × group interaction (*F*_1,49_ = 4.18, *p* = 0.046, *ηp*^2^ = 0.079) for PHQ-9 scores, where the probiotic group had significantly reduced depression ratings after 4 weeks (*F*_1,49_ = 6.60, *p* = 0.013, *ηp*^2^ = 0.119). This interaction remained after the inclusion of additional participants (*F*_1,69_ = 4.66, *p* = 0.034, *ηp*^2^ = 0.063) where the probiotic group had lower scores after supplementation (day 0: 12.37 ± 0.62 *v.* day 28: 6.57 ± 0.78; *F*_1,69_ = 6.83, *p* = 0.011, *ηp*^2^ = 0.09). There were no significant effects of placebo on PHQ-9 scores between day 0 and day 28. An analysis of individual items in the PHQ-9 from all participants (*n* = 71) revealed a main effect of group for question 4 (‘Feeling tired or having little energy’), where the subjects taking the probiotic scored less than placebo (placebo: 1.89 ± 0.1 *v*. probiotic: 1.49 ± 0.1; *F*_1,69_ = 8.03, *p* < 0.01). A time × group interaction was not detected. However, for item 7 (‘Trouble concentrating on things, such as reading the newspaper or watching television’), there was a significant time point × group interaction (*F*_1,69_ = 7.70, *p* = 0.007, *ηp*^2^ = 0.100), where the probiotic group scored less at 4 weeks compared to the beginning of the trial (day 0: 1.74 ± 0.16 *v*. day 28: 0.86 ± 0.16; *F*_1,69_ = 6.49, *p* = 0.013, *ηp*^2^ = 0.086).

**Table 2.** Subjective measures of mood, anxiety and affect

	Probiotic (*n* = 25)	Placebo (*n* = 26)	*p*
PHQ-9			
Day 0	12.01 (0.76)	12.65 (0.75)	0.046
Day 28	6.72 (0.90)	9.96 (0.88)	
STAI-T			
Day 0	52.56 (11.41)	56.85 (7.26)	0.917
Day 28	46.68 (11.27)	50.69 (11.19)	
STAI-S			
Day 0	44.92 (10.80)	46.77 (8.24)	0.188
Day 28	40.20 (10.75)	46.92 (13.53)	
PANAS (positive)			
Day 0	24.84 (8.67)	21.69 (7.42)	0.499
Day 14	26.40 (8.69)	25.45 (7.95)	
Day 28	25.32 (8.47)	25.04 (8.89)	
PANAS (negative)			
Day 0	17.68 (6.00)	19.92 (5.98)	0.780
Day 14	17.89 (7.09)	21.20 (7.05)	
Day 28	17.16 (4.85)	19.46 (6.95)	

PHQ-9, Patient Health Questionnaire-9; STAI, State/Trait Anxiety Inventory (-T: trait; -S: state); PANAS, Positive and Negative Affective Scale.

Data are presented as means ± (s.e.m.). Repeated measures analysis was performed with the group as a between-subjects factor (placebo, probiotic) and time-point (day 0, day 14, day 28) as a within-groups factor. The *p* values displayed are for the group × time-point interaction

There were no significant time point × group interactions (*F*_1,49_ < 2.0, *p* > 0.050) for either state or trait STAI scores (Table 2), but a main effect of time point for a trait (*F*_1,49_ = 20.96, *p* < 0.001). No significant treatment × time point interaction or main effect of the group were observed for PANAS positive scores, but there was a trend main effect of time point (*F*_2,96_ = 2.72, *p* = 0.073). For PANAS negative scores, there were no significant interactions though a trend main effect of group was seen, with the probiotic group scoring lower on the 3 time-points (*F*_2,96_ = 3.212, *p* = 0.079).

- Correlations

In the probiotic group, there were significant negative correlations between the reduction in PHQ-9 scores and percentage correct recall of both positive (Pearson *r* = −0.472, *p* = 0.017, *n* = 25) and negative (Pearson, *r* = −0.445, *p* = 0.026, *n* = 25) words in the EMEM. Thus, subjects who showed greater reductions in depression recalled fewer words with emotional meaning. No other correlations were observed.

## Discussion

We have tested whether a daily intake of a multispecies probiotic for 28 days reduced the processing of negative emotional cues and/or increased attention to positive emotional stimuli in volunteers with moderate depression. The present data demonstrate that subjects taking the probiotic were more accurate at recognising all facial expressions compared to those on placebo, and were less vigilant to both positive and negative stimuli. The probiotic group also displayed both decreased sensitivity to both win and loss outcomes in the PILT, and reduced learning in one component in the AVLT. A significant reduction in the subjective rating of depression was observed in the probiotic group, though there was a lack of direct correlations between emotional measures and mood scores. Finally, probiotic supplementation did not influence sleep or the concentrations of salivary waking cortisol or plasma CRP. Taken together these data suggest that the intake of this probiotic alters emotional processing in moderately depressed people in a different way to that previously reported with conventional antidepressant treatments.

According to a contemporary neuropsychological model, antidepressants work by reducing negative bias towards emotionally salient cues and/or increasing bias towards positive affective information (Godlewska & Harmer, 2021). In the present study, however, the probiotic group were more accurate in identifying all emotions in the FERT and reduced vigilance to both positive and negative cues in the dot-probe task, which suggests increased perception and attention towards non-emotional cues. The notion that the probiotic overall decreased the salience of emotional stimuli is consistent with our findings in other cognitive tasks.

In the PILT, the probiotic group were less likely to select both the high-probability wins and the low-probability losses, compared to the placebo group, which is indicative of reduced reward learning. Although this is inconsistent with antidepressants increasing win responses (Murphy et al., 2020; Walsh et al., 2018), it is in-keeping with the probiotic group being less responsive than placebo in both the dot-probe task (emotional perception) and a component of the AVLT (short-term verbal memory). However, an overall cognitive impairment is unlikely since the probiotic did not affect emotional (EMEM, EREC tasks) or implicit (priming task) memory, and improved item 7 scores (regarding concentration) of the PHQ-9. It is possible therefore, that the probiotic influenced the cognitive processes that discern emotion weighted outcomes in attention and learning. Indeed, the strong negative correlation between the reduction of PHQ-9 scores and correct recollection of both positive and negative words in the EMEM by the probiotic group, indicates that improved mood is associated with reduced memory of emotion-related words. Thus, the probiotic could be said to convey some degree of emotional impartiality where participants are less sensitive to specific emotional stimuli or are less influenced by losses and wins.

The susceptibility of the probiotic group to proactive interference in the AVLT, has been observed following a single administration of the benzodiazepine, lorazepam (Tsakonas, Kirkby, Montgomery, & Daniels, 1996). Similar to our current data, the drug reduced recollection of List B words but immediate and delayed recall remained unaffected. Lorazepam also led to mild deficits in two of the five List A recollection trials, though performance in subsequence trials returned to placebo levels. Building new associations in memory and impairing the acquisition of novel information is an accepted cognitive mechanism that underlies the anxiolytic effects of benzodiazepines (Griffin, Kaye, Bueno, & Kaye, 2013). Indeed, given the aforementioned similarity with benzodiazepines and based on animal studies, it is reasonable to suggest that the probiotic we used influenced central GABA neurotransmission.

Earlier work has demonstrated that the administration of the Lactobacillus strain, *L. rhamnosus* (JB-1) to mice, improved emotional behaviours and altered central GABA receptor gene expression and these effects were annulled by subdiaphragmatic vagotomy (Bravo et al., 2011). The intake of this probiotic has also been shown to increase the concentration of GABA itself in the mouse brain (Janik et al., 2016), and this might also be mediated by the vagus nerve as vagal stimulation has been reported to increased GABA concentrations in cerebrospinal fluid of people with treatment-resistant depression (Groves & Brown, 2005). The presence of *L. rhamnosus* in the current probiotic therefore, may have altered emotional processing, AVLT performance and/or mood through the modulation of the central GABA system, perhaps by influencing gut-brain vagus nerve activity. Parenthetically, transcutaneous auricular vagus nerve stimulation (taVNS) which is being trialled as a treatment for major depression, also reduces the intensity of responses to emotion-eliciting images in healthy volunteers (De Smet et al., 2021). The latter observation parallels our finding that the probiotic reduced vigilance to emotions in the dot-probe task, which also suggests an involvement of the vagus nerve in the actions of the current probiotic. Further pre-clinical studies are required to confirm whether the current probiotic modulates vagus nerve activity and/or GABA neurotransmission, and one approach to test the latter would be to repeat the present study with a non-convulsant GABA antagonist (Johnston, 2013) administered with the probiotic. However, it is notable that a change in anxiety was not observed which suggests that the mechanism may not be benzodiazepine-like, and may involve other neurotransmitters.

There is no doubt that gut microbiota also influences 5-HT and its metabolism: studies in germ-free mice and antibiotic administered rats show significant reductions in brain 5-HT in the absence (Clarke et al., 2013) or reduction (Hoban et al., 2016) of gut microbiota, respectively. Conversely, feeding mice a *B. longum* species, replenishes decreased levels of 5-HT following stress-induced behavioural despair (Tian et al., 2019). Based on these findings, it is tempting to suggest that subjects with moderate depression in the current study may have had a pre-existing deficit in central 5-HT levels which was restored by the *B. longum*, contained within the probiotic used. Indeed, further speculation may consider *B. longum* to have improved mood through 5-HT enhancement whereas the Lactobacilli in the probiotic, altered emotional processing through the GABA system. A probiotic that contains several species of bacteria may be expected to impart several effects on host neurochemical and physiological pathways, and so the impact of the current probiotic on central dopamine which is also influence by gut microbiota (González-Arancibia et al., 2019), cannot be ruled out. Further exploration into these possibilities although necessary, will be complex given that individual bacterial strains within a single species will have distinct properties.

A study by Savignac, Kiely, Dinan, and Cryan (2014) demonstrated the differential effects of two Bifidobacteria strains on emotional behaviours in mice. Thus, whilst both *B. longum* 1714 and *B. breve 1205* reduced anxiety in a stress-sensitive mouse strain, only *B. Longum* 1714 imparted an antidepressant-like effect. Antidepressant action of *B. longum* 1714 alone has not been observed in healthy volunteers (Moloney et al., 2020), and although *B. breve* has not been studied in mood disorders, it has been shown to reduce clinical ratings of anxiety and depression in schizophrenia (Yamamura et al., 2021). The *B. infantis* strain, which is also a constituent of the current probiotic, is reported to have improved mental well-being in people with inflammatory bowel syndrome (Ma et al., 2019). These studies not only demonstrate the strain-dependent effects of probiotics, but also illustrate that the antidepressant properties of probiotics have been reported when depression is comorbid with another condition. It is difficult, therefore, to pinpoint which species, or a combination thereof, in the current probiotic contributed to the changes in emotional processing and/or depression scores since bacteria with antidepressant action in comorbid depression, may not be effective in depression as a primary diagnosis. Our study recruited people with untreated, moderate depression in the absence of any other mental or physical illness, and so the present data inform on the bacterial strains that are most likely to influence emotional processing and mood in depressive illnesses that do not have somatic or other psychiatric origins.

Probiotics have been shown to improve sleep parameters (for review see Sen et al., 2021), and so these were also measured in the current investigation. Analysis of night-time actigraphy data revealed a significant group × total activity interaction, although follow up analysis showed that the increased total activity in the probiotic group after supplementation was not significantly different to the placebo. This is difficult to interpret as sleep restlessness after probiotic intake, given that the other sleep parameters did not change in either group after the intervention. Furthermore, the inspection of item 4 of the PHQ-9 regarding feelings of tiredness, indicated that subjects taking the probiotic felt less fatigued than those on placebo, which contradicts the possibility that the probiotic group experienced agitated sleep. Given that another probiotic improved sleep quality after a daily 24-week intake (Nishida et al., 2019), it is likely that a longer supplementation time is required to reveal any effects of the current probiotic on sleep and activity.

The present investigation has demonstrated that the probiotic did not significantly influence salivary CAR, which is in contrast to our earlier study of a prebiotic in healthy volunteers (Schmidt et al., 2015) and an antidepressant (Knorr et al., 2012). However, whilst one study confirms that a probiotic does not influence the secretion of cortisol in depression (Rudzki et al., 2019), others have demonstrated probiotic modulation of stress-induced cortisol release (Nishida et al., 2019; Wang et al., 2019). Furthermore, Nishida et al. (2019) administered their probiotic for 24 weeks. Therefore, probiotic effects on CAR may be dependent on the pre-existing levels of emotional stress, and/or the duration of supplementation, and so the influence of the current probiotic on cortisol secretion, cannot be entirely ruled out until further investigations are performed.

**Fig. 3.**
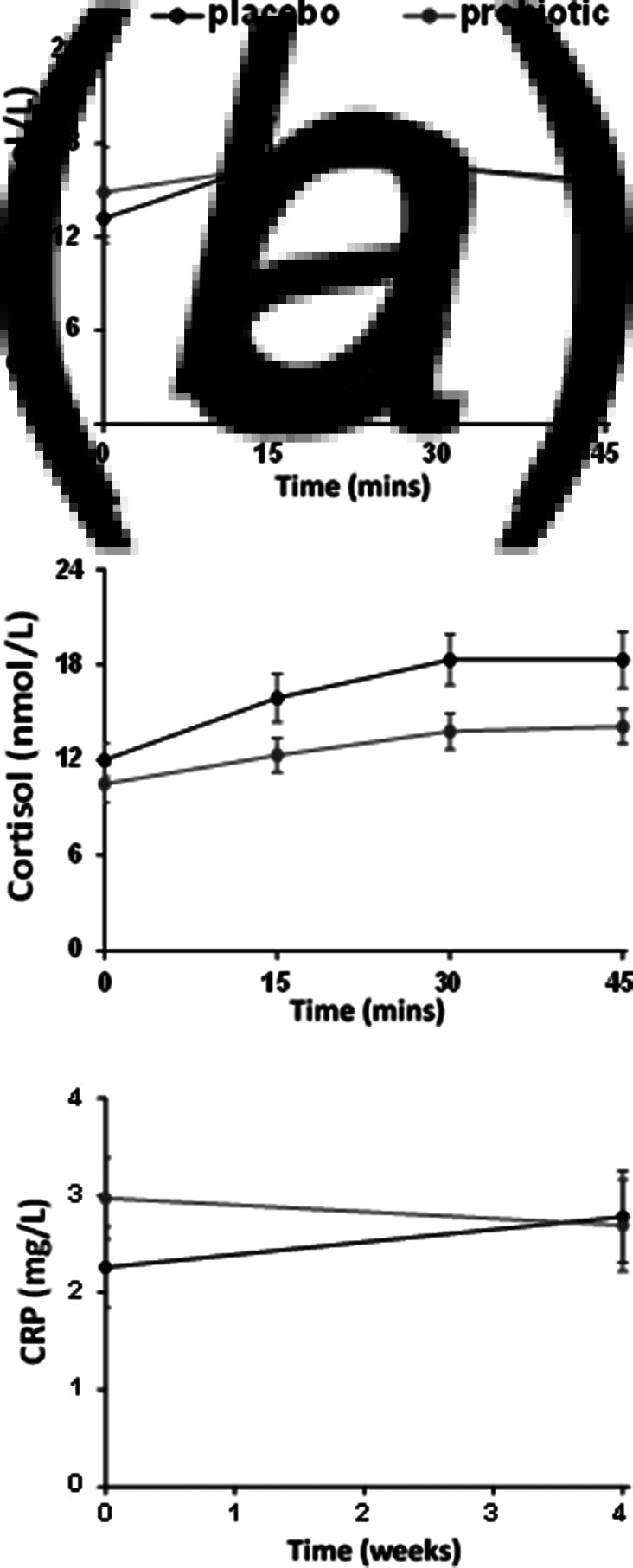
Salivary cortisol awakening response (CAR) and plasma CRP following placebo and probiotic supplementation. There were no significant differences in CAR, within and between probiotic (*n* = 35) and placebo (*n* = 36) groups at baseline (*a*) or after 28 days supplementation (*b*). No interactions or main effects of group on plasma CRP concentrations were observed following placebo (*n* = 26) or probiotic (*n* = 25) supplementation (*c*).

The unaltered concentrations of plasma CRP after supplementation contrasts the study of Akkasheh et al. (2016) who demonstrated reduced circulating CRP following probiotic intake in major depression, but is consistent with a report of a probiotic not influencing the levels of other circulating immune markers in the disorder (Rudzki et al., 2019). Of course, without having measured other immune factors, such as the interleukins, we cannot conclude that the unchanged CRP levels we observed, reflect an immune-independent mode of action for our probiotic. However, a similar argument to that offered to explain the lack of changes in CAR can also be applied to the CRP data. Low-grade inflammation is prominent in *major* depressive disorder (Beurel, Toups, & Nemeroff, 2020), and in animal studies, the anti-inflammatory action of probiotics has mainly been demonstrated in models of stress (Moya-Pérez et al., 2017) or endotoxin-induced anxiety (Murray et al., 2019). Therefore, it is possible that the moderately depressed subjects we recruited may not have had an immune component to their condition as do some patients with major depression, and so in the absence of pre-existing inflammation, an effect of the probiotic on the immune system could not be detected. The relatively low number of participants in each group, and high variability of circulating CRP concentrations (Fig. 3*c*, online Supplementary Table S4) potentially masking an effect of the probiotic, should also be considerations.

Several limitations of the current investigation are worth noting. First, subjects did not perform emotional and cognitive tasks at baseline, which precluded the analysis of within-subject effects of this probiotic. This was so that familiarity with the psychometric tests did not influence the participants' performance of these tasks at the end of the study, the most crucial time point (Harmer et al., 2011). Second, the intervention was only for four weeks, whereas other probiotic studies reporting changes in sleep and anxiety, have been longer. The relatively short duration of the present study was based on another multi-species probiotic investigation (Steenbergen, Sellaro, van Hemert, Bosch, & Colzato, 2015) and our study of emotional processing with a prebiotic (Schmidt et al., 2015). In these instances, the aim was to detect early changes in emotional processing prior to alteration in a mood so that causal mechanisms rather than an effect of a mood change could be revealed. Third, without an antidepressant comparator, it is not known for certain whether the observed changes in emotional processing after the probiotic supplementation were indeed distinct from the effects of conventional medication in our particular cohort of subjects, and duration of treatment. Follow up studies of probiotics and emotional processing in moderate depression should include, therefore, an antidepressant group, and perhaps perform emotional and cognitive tasks earlier than 4 weeks to test if the observed changes in the current study were preceded by alterations that are similar to those reported other interventions.

A final potential limitation to consider is the absence of data on participant gut microflora. That is, the probiotic may have conveyed a psychotropic effect by altering the constitution of intrinsic gut bacterial communities. At the conception and design of the study, the authors were guided by a systematic review that demonstrated the lack of effect of probiotics on faecal microbiota composition in healthy volunteers (Kristensen et al., 2016). One of the issues that was proposed to have contributed to this result was the small sample sizes and lack of statistical power in the seven eligible randomised controlled trials reviewed, which comprised of 20–81 participants in the probiotic and placebo groups. This is likely to have been a caveat in a more recent study that also did not show changes in the gut microbiome of people with major depression following an 8-week supplementation with a probiotic or placebo (34 and 37 participants, respectively), following within-subject analysis and compared to healthy controls (Chahwan et al., 2019). According to power calculations based on our primary outcome (see Methods), we required 25 participants per group which was within the range considered to be underpowered for a meaningful interpretation of faecal genomic data (Kristensen et al., 2016). In view of this and the potential added burden to participants, faecal samples were not collected for analysis. Future probiotic intervention studies therefore, require greater sensitivity to detect changes in the gut microbiome, which can be achieved by recruiting participant numbers similar to those that have demonstrated an altered abundance of bacterial species in subjects with major depression (Valles-Colomer et al., 2019).

Overall, our data show that the repeated intake of multispecies probiotic changes emotional processing in people with moderate depression, in ways that are different from those seen with contemporary antidepressant therapies in healthy volunteers and in subjects with a major depressive disorder. This may indicate the involvement of a different neurotransmitter system or the modulation of groups of pathways that are sensitive to peripheral signals. The decision to recruit people with moderate depression was made on the assumption that they would have a pre-existing aberration in emotional processing (based on earlier work in major depression), so that any effect of the probiotic on the latter could be readily detected. Although correlations between any changes in emotional processing and mood ratings were part of the a priori analytical plan, an improvement in depression scores was not an intended outcome of the study, but nevertheless, a decrease in depression scores was observed. These observations may suggest therefore, that probiotic administration may be an ‘early intervention’ strategy to reduce the risk of people with mild to moderate depression developing a major depressive disorder. Further work is required to test the duration of the beneficial effects of the current probiotic at the levels of emotional processing, mood and metabolism.

## Supplementary material

For supplementary material accompanying this paper visit https://doi.org/10.1017/S003329172100550X. click here to view supplementary material
