# Supplementary material for: Multispecies probiotic administration reduces emotional salience and improves mood in subjects with moderate depression: a randomised, double-blind, placebo-controlled study
Source: Psychol Med. 2022 Feb 7;53(8):3437–47. doi: 10.1017/S003329172100550X (PMC10277723; doi:10.1017/S003329172100550X)
Supplement: Supplementary file 1 [file S003329172100550Xsup001.docx]

**SUPPLEMENTARY INFORMATION**

**Fig S1.** Recruitment flow diagram (based on CONSORT)

***Supplementary Methods***

Randomization and blinding

Randomisation was performed by a qualified researcher not involved in the study using the dedicated (free) software sealed envelope (<https://www.sealedenvelope.com>). The randomisation programme included a minimisation algorithm to ensure balanced allocation of participants across the treatment groups by sex, and used a block design of 4. Allocation ratio was 1:1 for treatment (probiotic vs placebo) and 1:1 for sex (male and female).

The code was stored in a locked filling cabinet in the Neurosciences Building at the Department of Psychiatry, Warneford Hospital, Oxford, England. Both probiotic and placebo were encapsulated using identical casings, and the individual single doses were stored in sealed envelopes labelled with the corresponding study code. All researchers involved in the study remained blind to this code throughout the entire study duration (May 2017 to February 2018) and data analysis (after February 2018). The study stopped when the sample size was reached (N=40). Unblinding only occurred after data analysis was completed. All the study sessions were conducted at the Department of Psychiatry, Warneford Hospital, Oxford, England.

Emotional processing test battery (ETB) methods

*Facial Emotional Recognition Task (FERT):* Images of faces displaying 6 basic emotions (happy, angry, surprised, sad, fearful and disgusted) were flashed onto a black screen for 500ms. These varied in intensity by 10% increment, 0% being neutral and 100% being the full expression, giving 250 images per test over four trials. Participants categorised expressions by pressing the corresponding keyboard key. Measures were accuracy of classification, reaction time and bias in misclassifying faces.

*Emotional Categorisation Task (ECAT):* A series of personality adjectives were presented on screen for 500ms, half with a positive valence (i.e. friendly, thoughtful, gracious) and half negative (i.e. dull, untidy, manic) (each trial with 60 words, 30 of each valence). Participants were asked to categorise words as ‘likeable’ or ‘dislikeable’ by pressing a computer key. Specifically, participants were asked to imagine whether they would like or dislike overhearing someone referring to them as possessing this characteristic, so that the judgment was in part self-referential. Words were matched for length, frequency of use in the English language and clarity. Outcome measures were accuracy, reaction time relative to valence.

*Dot Probe Task:* Participants were presented with pairs of vertically consecutive faces on screen, one neutral and the other either neutral, happy or fearful; order of pairs was randomized. A pair of dots appeared after the faces in one of the two positions. Participants classified the dot orientation as either vertical (:) or horizontal (..) by pressing keyboard keys. Each of the 3 possible pairs of faces were presented 33 times, giving a total of 99 trials, in one of two conditions: masked and unmasked. In the unmasked condition, the face pair was presented for 100ms and then a probe appeared in the location of one of the preceding faces. In the masked condition, the face pair was displayed for a short period of time (16ms) and was immediately replaced by a mask consisting of a jumbled face and displayed for 84ms, before being replaced by a probe (the dot). In ‘congruent’ trials, dots replaced the happy or fearful face, while in ‘incongruent’ trials they replaced the neutral face. The outcome measure was *attentional vigilance scores*, which were calculated for each participant by subtracting the mean reaction time from trials when probes appeared in the same position as the emotional face (congruent trials) from trials when probes appeared in the opposite position to the emotional face (incongruent trials).

*Emotional Recall Task (EREC) and Emotional Recognition Memory Task (EMEM):* After the Dot Probe (i.e., approximately 12 mins after the ECAT), participants were asked to write down as many words as they could recall from the ECAT task, regardless of valence. Outcomes were accuracy and false-positive recollections relative to valence. A computerised recall task followed free-recall. Words were displayed on screen for 500ms; they contained the 60 original ECAT words and 60 distracter words, 30 of each valence. Participants categorised the word as ‘familiar’ or ‘unfamiliar’ by pressing corresponding keyboard keys. Outcome measures were accuracy and reaction times relative to valence (EREC and EMEM) and intrusions (EREC).

Probabilistic Instrumental Learning Task (PILT)

In this task, participants were presented with two symbols at a time and instructed to pick the symbol they believed was most likely to result in a win (or least likely to result in a loss) with the aim of maximising their monetary pay off (Walsh et al, 2018). Participants completed three runs of the task, with each run consisting of 60 trials split into 30 “win” trials, where participants could either win 20p or 0p and 30 “loss” trials, in which participants could either lose 30p or 0p. Across a run, one pair of stimuli were used for win trials and one for loss trials, with the stimuli used changing between runs. One symbol in a pair had a 0.7 probability of being followed by the win/loss outcome and a 0.3 probability of being associated with the no-change outcome. The other symbol had reciprocal probabilities. After participants made a choice, feedback on the trial outcome was presented.

Priming Task

This task measured implicit memory, where prior exposure to a stimulus influences responses to the next stimulus. Participants were asked to locate an “X” that would appear at one out of 4 possible locations on the screen, and press a corresponding location key on the keyboard. A distracter “O” would also be shown at a second location. Each trial starts with the presentation of a fixation cross for around 1500 ms, followed by a priming display. Participants will then be presented with a second shorter fixation cross for around 400 ms, and finally a probe display. Participants will have to respond to both the priming and probe display, and both displays remain on screen until a response is recorded. The locations of the “X” and “O” within the two displays in a trial result in 4 conditions – 2 experimental conditions (repetition priming and negative priming), and 2 control conditions (imbalance and control). There are a total of 192 trials (48 trials per condition), presented to participants in 3 blocks with a 15 second break in between blocks. Data was analysed by calculating difference scores from reaction times from the 2 experimental conditions compared to the control condition. Only trials with correct responses to the prime display were included in the analysis.

Auditory Verbal Learning Task (AVLT)

In the AVLT, participants were read a list of nouns (List A) and asked to immediately verbally recall it 5 times. An unrelated list of words was then presented (List B) and participants were again asked to recall them. This was an interference trial from which ‘proactive interference’ could be evaluated (Tsakonas et al, 1996). Participants were then asked to recall List A immediately (short-delay) and after a delay of 15 min (long-delay). Participants then completed a recognition task where they were required to indicate which of a list of words (15 List A words, 35 distractors) had previously been presented. Number of correct words were measured in all trials.

Sleep measures

Actigraphy was used to provide an objective measurement of sleep. Participants wore a MotionWatch 8 device (MW8; CamNtech Ltd, Cambridge, U.K.). These telemetric devices are worn on the non-dominant wrist, using movement to determine rest/activity cycles. Data were recorded at 30-s epochs in accordance with the validated sleep algorithm, and analysed using the inbuilt sleep software. Students were asked to press an event marker button on the device when they were in bed, had switched off the lights, and were attempting to sleep, and again when they woke up in the morning and had no intention of returning to sleep. As actigraphy uses movement to determine sleep onset/offset, this method was considered the most accurate way to differentiate sleep intention from, for example, lying in bed using social media. Each sleep period over the 7 days (first and last weeks of supplementation) was manually evaluated. The markers for ‘fell asleep’ and ‘woke up’ (final awakening) were automatically adjusted according to the event markers and the activity data. If the subject forgot to press the event marker, the sleep diary time was used instead, and if there was no corresponding diary day, or activity was significantly discrepant from the event marker or diary, then the markers were placed when activity ended or began (in this instance, sleep latency data were consequently not included). The sleep variables were calculated separately for weekday and weekend and averaged over the number of nights provided to yield habitual sleep patterns. These included ‘Time you went to bed’ (time the participant put the lights out and tried to sleep) ‘Time you woke up’ (time the participant woke up at the end of the main sleep period and had no intention to sleep longer), sleep onset latency (time between going to bed and falling asleep), total sleep time (total time spent in sleep according to the epoch-by-epoch wake/sleep categorisation), wake after sleep onset (total time spent in wake according to the epoch-by-epoch wake/sleep categorisation), and sleep efficiency (total sleep time expressed as a percentage of time in bed after repose and getting up).

**Fig S2.**  Participants completed a food and sleep diary in the first and last seven days of the trial. Changes in the amount each item consumed before and after placebo or probiotic supplementation, was analysed using a linear fixed effect model on the weekly average of each item.

***Supplementary Results***

| TEST | Placebo (n=26) Probiotic (n= 25) |
| --- | --- |
| Emotional Task Battery |  |
| FERT:  Accuracy (%)*  Neutral  Anger  Disgust  Fear  Happy  Sad  Surprise  Reaction time (ms)  Neutral  Anger  Disgust  Fear  Happy  Sad  Surprise  Misclassifications (%)  Neutral  Anger  Disgust  Fear  Happy  Sad  Surprise | 68.84 (3.02) 79.20 (3.08)  45.96 (2.24) 47.80 (2.29)  40.67 (3.06) 44.40 (3.10)  45.67 (3.07) 50.10 (3.14)  64.04 (1.91) 63.90 (1.95)  43.94 (3.02) 49.80 (3.08)  53.55 (2.21) 58.20 (2.26)  1600 (96.4) 1585 (98.3)  1806 (95.1) 1881 (97.0)  1935 (117.5) 1933 (119.9)  1913 (108.6) 1951 (110.8)  1579 (52.7) 1546 (53.7)  1781 (84.2) 1719 (85.9)  1815 (97.6) 1947 (99.6)  66.96 (4.12) 66.61 (4.12)  14.03 (1.57) 13.30 (1.57)  11.80 (1.56) 10.26 (1.56)  10.34 (1.26) 6.80 (1.26)  6.46 (2.02) 1.96 (2.02)  7.84 (1.24) 8.26 (1.24)  12.9 (1.79) 8.57 (1.79) |
| ECAT:  Accuracy (%)  Positive  Negative  Reaction time (ms)  Positive  Negative | 96.13 (1.30) 95.73 (1.30)  94.93 (1.28) 96.00 (1.28)  983.55 (40.85) 942.26 (40.85)  1032.6 (43.37) 1014.0 (43.37) |
| EREC:  Accuracy (%)  Positive  Negative  Intrusions (%)  Positive  Negative | 56.95 (2.90) 53.303 2.967  43.04 (2.90) 46.697 2.967  51.62 (8.11) 49.733 8.276  29.14 (7.37) 38.267 7.517 |
| EMEM:  Accuracy (%)  Positive  Negative  Intrusions (%)  Positive  Negative | 56.95 (2.90) 53.30 (2.96)  43.04 (2.90) 46.69 (2.96)  51.62 (8.11) 49.73 (8.27)  29.14 (7.37) 38.26 (7.51) |

**Table S1**. Summary of data from the emotional test battery and priming task (cognitive test) performed by participants on the last day of placebo or probiotic supplementation (day 28). Data are presented as means +/- (SEM). *p<0.05 overall effect of group (see Fig 2 in main article).

**Table S1 (continued)**

| TEST | Placebo (n=26) Probiotic (n= 25) |
| --- | --- |
| Emotional Test Battery |  |
| Dot-probe task  Unmasked vigilance (relative to neutral, ms)  Happy  Fear  Masked vigilance (relative to neutral, ms)  Happy  Fear | 10.29 (6.52) -4.16 (6.65)  -2.75 (7.16) -12.86 (7.30)  10.29 (6.52) -2.75 (7.16)  -4.16 (6.65) -12.86 (7.30) |
| Priming Task  Priming effect (ms):  Negative priming  Repetitive priming | 5.86 (5.54) 15.64 (5.89)  38.23 (11.49) 42.68 (12.21) |

| Sleep parameter | Placebo | | Probiotic | |
| --- | --- | --- | --- | --- |
|  | *W1 (n=26)* | *W4 (n=25)* | *W1 (n=25)* | *W4t (n=24)* |
| Actual sleep time (s) | 24648 (390) 24168 (380) | | 24068 (901) 24719 (420) | |
| Sleep efficiency (%) | 86.0 (1.0) 85.9 (0.9) | | 82.6 (3.2) 84.3 (1.0) | |
| Sleep latency (s) | 646 (134) 545 (95) | | 658 (98) 705 (126) | |
| Wake bouts (s) | 40.4 (2.5) 39.3 (2.2) | | 36.6 (2.3) 39.9 (1.8) | |
| Mean wake bouts (s) | 81.7 (2.7) 82.7 (3.3) | | 83.4 (4.5) 90.2 (4.1) | |
| Immobility (mins) | 429 (6.6) 422 (6.8) | | 420 (15.9) 436 (7.0) | |
| Mean activity/epoch (s) | 4.76 (0.35) 4.60 (0.27) | | 4.28 (0.53) 5.41 (0.42) | |
| Total activity (s) | 4577 (393) 4257 (302) | | 4368 (313) 5112 (401)* | |

**Table S2**. Sleep parameters measured by actigraphy in the first (*W1*) and forth (*W4*) week of daily supplementation with placebo or probiotic. Data are presented as means +/- (SEM) *p<0.05, group x total activity interaction.

| Food Item | Placebo | | Probiotic | |
| --- | --- | --- | --- | --- |
|  | *W1 (n=26)* | *W4 (n=25)* | *W1 (n=25)* | *W4 (n=24)* |
| Dairy | 1.44 (0.19) 1.49 (0.20) | | 1.41(0.19) 1.41 (0.21) | |
| Cereals | 2.0 (0.16) 2.25 (0.16) | | 2.0 (0.17) 2.24 (0.15) | |
| Vegetables | 2.10 (0.2) 1.80 (0.22) | | 2.48 (0.30) 2.14 (0.18) | |
| Fruit | 1.58 (0.17) 1.49 (0.23) | | 1.55(0.18) 1.43(1.8) | |
| Protein | 1.73 (0.11) 1.69 (0.14) | | 1.79 (0.13) 1.62 (0.12) | |
| Sweets | 1.37 (0.15) 1.25 (0.11) | | 1.14 (0.13) 1.06 (0.10) | |
| Alcohol | 0.85 (0.21) 1.04 (0.25) | | 0.84 (0.2) 0.63 (0.10) | |
| Water | 4.59 (0.57) 4.33 (0.76) | | 5.42 (0.53) 4.88 (0.47) | |
| Coffee/Tea | 2.29 (0.33) 2.02 (0.31) | | 2.21 (0.22) 2.13 (0.22) | |

**Table S3**. Food items consumed in the first (*W1*) and fourth (*W4*) week of a daily supplementation with placebo or probiotic. Data are presented as means +/- (SEM)

| Biomarker (concentration) | Placebo | | Probiotic | |
| --- | --- | --- | --- | --- |
|  | *Day 0 (n=36)* | *Day 28 (n=36)* | *Day 0 (n=35)* | *Day 28 (n=35)* |
| Salivary Cortisol 0’ | 12.90 (1.29) 12.30 (1.08) | | 13.80 (2.20) 10.20 (1.05) | |
| (nmol/L) 15’ | 15.30 (2.13) 16.20 (1.17) | | 15.30 (2.25) 12.90 (0.99) | |
| 30’ | 16.20 (1.17) 17.70 (1.26) | | 15.90 (1.35) 14.10 (1.07) | |
| 45’ | 14.70 (1.20) 17.40 (1.59) | | 14.70 (0.91) 13.50 (0.93) | |
|  | *Day 0 (n=26)* | *Day 28 (n=26)* | *Day 0 (n=25)* | *Day 28 (n=25)* |
| Plasma CRP (mg/L) | 2.26 (0.42) 2.78 (0.47) | | 2.97 (0.42) 2.69 (0.47) | |

**Table S4**. Concentrations of salivary cortisol and plasma C-Reactive Peptide (CRP) in participants at the start (Day 0) and the end (Day 28) of daily, probiotic or placebo supplementation. Subjects provided saliva samples after waking (0’) and subsequent samples every 15 minutes for 45 minutes. Blood was taken by investigators when subjects visited the research facility on Days 0 and 28. Data are presented as means +/- (SEM)
